# Clinical Outcomes of Older Patients with Non-Variceal Upper Gastrointestinal Bleeding Taking Anti-Thrombotic or Non-Steroidal Anti-Inflammatory Agents

**DOI:** 10.5152/tjg.2023.23226

**Published:** 2023-09-01

**Authors:** Muhammed Bahaddin Durak, Cem Şimşek, İlhami Yüksel

**Affiliations:** 1 Department of Gastroenterology, Ankara City Hospital, Ankara, Turkey; 2 Department of Gastroenterology, Health Sciences University, Mehmet Akif İnan Training and Research Hospital, Şanlıurfa, Turkey; 3 Graduate School of Health Sciences, Hacettepe University, Ankara, Turkey; 4 Department of Gastroenterology, Ankara Yıldırım Beyazıt University Faculty of Medicine, Ankara, Turkey

**Keywords:** Upper gastrointestinal bleeding, anti-thrombotic drugs, non-steroidal anti-inflammatory drugs

## Abstract

**Background/Aims:**

Non-variceal upper gastrointestinal bleeding is a well-established complication of non-steroidal anti-inflammatory drugs and anti-thrombotics. Both medication groups are frequently used by older populations and increase the incidence of non-variceal upper gastrointestinal bleeding; however, their impact on etiology and outcomes of non-variceal upper gastrointestinal bleeding has not been well defined. We aimed to compare the etiology and outcomes of non-variceal upper gastrointestinal bleeding in older patients who use anti-thrombotics and non-steroidal anti-inflammatory drugs or do not use either of them.

**Materials and Methods:**

This is a single-center prospective study of patients older than 65 years with non-variceal upper gastrointestinal bleeding. Endoscopic findings, laboratory values, blood transfusion, endoscopic treatment, re-bleeding, and 30-day mortality rates were recorded.

**Results:**

A total of 257 patients (median age 77.7 ± 8.2, 59% male) were included. Re-bleeding occurred in 25 (10%) and the 30-day mortality rate was 40 (16%). There was no statistically significant difference between patients using anti-thrombotics, non-steroidal anti-inflammatory drugs or non-users for blood transfusion (*P* = .46), endoscopic hemostasis (*P* = .39), re-bleeding (*P* = .09), and 30-day mortality (*P* = .45). Peptic ulcer was the most common etiology in all groups (124, 48%). Although the incidence of peptic ulcer was similar between drug users and anti-thrombotic users (*P* = .75), the incidence of peptic ulcer was significantly higher in patients using non-steroidal anti-inflammatory drugs than in patients who did not use drugs (*P* = .05). When the patients were analyzed as using anti-thrombotic drugs or non-steroidal anti-inflammatory drugs or neither, no statistically significant difference was found between ulcer location, ulcer number, and ulcer size.

**Conclusion:**

Non-variceal upper gastrointestinal bleeding increasingly occurs in older populations with several comorbidities; non-steroidal anti-inflammatory drugs or anti-thrombotics do not seem to change the clinical outcomes among older patients with non-variceal upper gastrointestinal bleeding.

**Main Points**

- There was no statistically significant difference in terms of clinical outcomes among older patients on anti-thrombotics or non-steroidal anti-inflammatory drugs (NSAIDs) and patients who do not use either anti-thrombotics or NSAIDs.
- The results suggest that the use of NSAIDs or anti-thrombotics in older patients does not impact the outcomes of non-variceal upper gastrointestinal bleeding.
- While the prevalence of upper gastrointestinal bleeding in the older patient population is increased by the use of NSAID and anti-thrombotics, the severity of clinical outcomes is unaffected.

## Introduction

Anti-thrombotic medications include those that inhibit platelet aggregation (anti-platelets), inhibit the formation of fibrin strands (anticoagulants), and dissolve existing clots (fibrinolytics). Anti-platelets including low-dose aspirin (LDA) and adenosine diphosphate (ADP) receptor antagonists are used for the prevention of cardiovascular and cerebrovascular diseases.^1^ Dual anti-platelet therapies (DAPT), mostly of LDA and clopidogrel combination, have become the standard of care for preventing stent thrombosis implantation of drug-eluting coronary artery stents.^2^ Anticoagulant medications are the mainstay of therapy for venous thromboembolism and for thromboprophylaxis in valvular heart disease and atrial fibrillation. Direct oral anticoagulants (DOACs; also called non-vitamin K oral anticoagulants) are oral medications that directly inhibit specific enzymes in the coagulation cascade. Two factors targeted by available DOACs are thrombin (factor IIa) and factor Xa. Dabigatran is the only oral direct thrombin inhibitor available for clinical use. Rivaroxaban, apixaban, edoxaban, apixaban, edoxaban, and betrixaban are oral direct factor Xa inhibitors.^3^

Anti-thrombotic agents, which include LDA, anti-platelets, and anticoagulants, are known to increase the risk of upper gastrointestinal bleeding (UGIB), with an estimated annual risk of 1.5%-4.5%.^4,5^ Non-steroidal anti-inflammatory drugs (NSAIDs) are another medication group well known to increase the risk of UGIB where non-selective NSAIDs increase the risk 4-fold and COX-2 inhibitors increase the risk 3-fold.^6,7^ Importantly, both anti-thrombotics and NSAIDs are frequently used by older populations given the increased prevalence of cardiovascular and joint diseases, further increasing the UGIB risk. In addition, old age alone is associated with a higher incidence of UGIB and worse clinical outcomes.^5-7^ Although the increased incidence of UGIB with anti-thrombotics and NSAIDs has been well described, their use in young and older patients may cause different clinical outcomes. Recent studies have demonstrated that anti-thrombotic therapy, use of NSAIDs, and old age are independent risk factors for UGIBs.^6,7^

Clinical characteristics and outcomes of UGIB in older populations using NSAIDs and anti-thrombotics need to be well understood. Although an increased incidence of UGIB during the administration of anti-thrombotic therapies has been extensively described, the risk of bleeding due to the use of anti-thrombotic agents and NSAIDs may cause different clinical outcomes in young and older patient groups. The precise characteristics of UGIB associated with anti-thrombotic therapies are less understood than those of UGIB unassociated with anti-thrombotic therapies, especially in older patients’ groups. In this prospective cohort study, we aimed to compare the endoscopic findings and clinical outcomes of UGIB in anti-thrombotic users and NSAID non-users presenting with UGIB.

## Materials and Methods

### Study Population

This is a prospective cohort study of older (age >65) patients who presented with non-variceal UGIB to the emergency department of a tertiary referral center between February 2019 and February 2020. All UGIB was confirmed with an upper endoscopy. Patients with variceal bleeding, who use both NSAIDs and anti-thrombotic agents were excluded from the study. The study population was separated into 3 groups according to the medication use at presentation: patients on anti-thrombotics (LDA, anti-platelets, anti-coagulants), patients on NSAIDs, and patients who did not use either anti-thrombotics or NSAIDs, who were defined as controls. Written informed consents were obtained from every patient before enrollment. The study was approved by the Institutional Review Board of Ankara City Hospital, Ankara, Turkey (E1/22/2951).

### Diagnosis and Management

Diagnosis of UGIB was first based on patients’ presentations, including coffee ground vomiting, hematemesis, melena, or blood in the nasogastric aspirate. For initial medical management, intravenous proton pump inhibitor therapy was promptly initiated. A blood transfusion was performed when hemoglobin levels were less than 8 g/dL. In patients with unstable hemodynamic status, signs of ongoing bleeding, or decreasing hematocrit despite transfusion, an endoscopy was performed in the first 12 hours. For clinically stable patients without evidence of severe bleeding, an endoscopy was performed within the first 24 hours, if possible, to guide management. Endoscopic treatment was performed when high-risk stigmata of recent hemorrhage were encountered, such as active bleeding (spurting /oozing) or non-bleeding visible vessels. Endoscopic treatment included thermal contact, mechanical therapy, or a combination of both, but not adrenaline alone. In case of endoscopic treatment failure, patients were consulted to interventional radiology or surgery. The decisions to discharge the patients were made based on the initial presentation, clinical course, and endoscopic findings. All patients were followed up for 30 days. Based on the available guidance from the European Society of Gastrointestinal Endoscopy, anti-thrombotic agents were restarted as soon as bleeding control was achieved.^8^ Patients who were discharged within 24 hours were followed up with outpatient clinic visits at week 1 and week 4.

### Data Collection and Study Outcomes

At the initial presentation, medical history, medication lists, laboratory results, and vital signs were recorded. Endoscopic data including the cause of bleeding, presence of ulcer, its location, number, size, and Forrest classification as well as the type of endoscopic treatments were collected. During admission, the need for transfusion, need for endoscopic treatment, re-bleeding, and need for interventional radiology or surgery consultations were recorded. Re-bleeding was defined as a decrease of hemoglobin of more than 2 g/dL with signs of active bleeding. In case of hemoglobin drop, a second look endoscopy was performed to assess re-bleeding. Survival was assessed as inpatient mortality and 30-day mortality. Clinical outcomes were defined as the need for blood transfusion, need for endoscopic hemostasis, need for interventional radiology/surgery, and the presence of re-bleeding, hospitalization, and 30-day mortality.

### Statistical Analysis

The distributions of continuous variables were investigated with analytic methods (Kolmogorov–Smirnov test). Continuous variables were presented using means and SD or medians with interquartile range according to the distribution pattern. The chi-square test was used to compare ulcer location (esophageal/peptic/gastric/duodenal ulcer), Forrest classification, erosive esophagitis, upper gastrointestinal malignancy, erosive/hemorrhagic gastropathy-duodenopathy, angioectasia, and dieulafoy lesion between groups. Also, the relationship between the size/number of ulcer and mortality/re-bleeding were analyzed using the *χ* ^2^ test. A 2-sided *P* < .05 was considered significant. All data were analyzed using Statistical Package for the Social Sciences software version 22 (IBM Corp.; Armonk, NY, USA).

## Results

During the study period, 578 patients with acute UGIB presented to the emergency department. Of 578, 321 patients were excluded as 209 were younger than 65 years, 54 had variceal bleeding, 38 refused endoscopy, 17 had missing data, and 3 used a combination of NSAIDs, anti-platelets, or anticoagulants. The final study population consisted of 155 (60%) patients on anti-thrombotics, 26 (10%) patients on NSAIDs, and 76 (30%) patients who were not using either anti-thrombotics or NSAIDs. Among anti-thrombotic users, 90 (35%) were on LDA, 8 (3%) were on DAPT, and 57 (22%) were on anticoagulants. Patients included in the study and the number of patients in each group are summarized in the flowchart.

Population median age was 77.7 ± 8.2 years, and 59% of patients were male. There was no significant difference in terms of comorbidities between patients on anti-thrombotics, NSAIDs, and controls. A previous history of UGIB was present in 56 (22%) of patients. For all cohorts, the baseline value for hemoglobin was 9.3 (±2.7) g/dL, BUN was 92.0 (55.5-141.0) mg/dL, and albumin was 34.5 (±6.7). Patients’ demographics, comorbidities, and clinical outcomes are summarized in Table 1.

Of 257 included patients, 64 (25%) were discharged from the emergency department within 24 hours, whereas 193 patients (75%) were admitted with a median duration of 5.0 (0-11.0) days. Hospitalization, internal care unit submission, and length of stay were statistically similar in the group using and not using anti-thrombotic agents. Transfusion was required in 167 (65%) cases. Endoscopic treatment was performed in 78 (30%) patients. Endoscopic therapy was successful in 83.3% of no used drugs while in 95.9% of using anti-thrombotics. No statistically significant difference was observed in both groups (*P =* .08). Re-bleeding occurred in 25 (10%) patients. Seven (3%) patients required interventional radiology or surgical treatment. Thirty-day mortality rate of the study population was 40 (16%). When patients on anti-thrombotics, NSAIDs, and controls were compared, hemoglobin and BUN levels on admission were not different between the 3 groups. Patients using NSAIDs and anti-thrombotics had similar clinical outcomes when compared to controls, including need for transfusion (*P* = .46, *P* = .27), need for endoscopic therapy (*P* = .39, *P* = .29), re-bleeding (*P* = .09, *P* = .78), and 30-day mortality (*P* = .45, *P* = .051) rates.

Endoscopic findings are summarized in Table 2. The cause of bleeding was peptic ulcer in 124 (48%) patients and it was the most common cause in all 3 groups. The frequency of peptic ulcer was significantly higher in patients on NSAIDs when compared to controls (*P* = .05) but was similar between anti-thrombotic users and controls (*P* = .75). Features of peptic ulcer—location, number, and size—were similar between the 3 groups. Patients on NSAIDs, but not anti-thrombotics, had more frequent Forrest II-c and III ulcers than controls (*P* = .01).

Two patients developed serious thromboembolic events during discontinuation of anticoagulants. The first patient was on warfarin for valvular heart disease, which was discontinued at the time of presentation, and complicated by cerebral infarction on the fourth day of hospitalization despite an INR of 2.1. The second patient was also on warfarin due to a history of deep vein thrombosis and was complicated by pulmonary thromboembolism on the seventh day of drug discontinuation.

## Discussion

In the present study, there was no statistically significant difference in terms of clinical outcomes including the need for blood transfusion, re-bleeding, hospitalization, need for interventional radiology/surgery, and 30-day mortality rates among older patients on anti-thrombotics, NSAIDs, and patients who do not use either anti-thrombotics or NSAIDs. And also, there was no statistically significant difference in age, hemoglobin, and BUN levels among the 3 groups. In all groups, the most common cause of UGIB was peptic ulcer. Non-steroidal anti-inflammatory drug users had the highest frequency of peptic ulcers but more of these ulcers were lower risk (Forrest II-c and III). Previous studies demonstrated that anti-thrombotic therapy, use of NSAIDs, and old age are each independent risk factors for UGIB.^3,9,10^ Although the increased frequency of non-variceal upper gastrointestinal bleeding (NVUGIB) due to NSAIDs and anti-thrombotic use is previously known,^11-13^ their impact on the prognosis of NVUGIB in older patients is not well defined. To our knowledge, this is the first study to evaluate the characteristics and outcomes of NVUGIB in older patients using anti-thrombotic or NSAIDs. Our results suggest that the use of NSAIDs or anti-thrombotics in older patients does not impact the outcomes of NVUGIB.

When compared to previous studies evaluating UGIB with NSAIDs or anti-thrombotics, our adverse outcome rates were higher including the need for transfusion (65%), endoscopic treatment (30%), hospitalization (75%), need for interventional radiology/surgery (3%), re-bleeding (10%), and 30-day mortality (16%).^14-19^ However, age can be a confounder as the included populations were general adults in these previous studies,^9,14,20^ whereas all patient groups in our study have the older age. In addition, approximately half of the population was on anti-thrombotics or NSAIDs, less than 70% frequency in our population. In patients with UGIB under anti-thrombotics, previous studies by Yamaguchi et al^16^ and Nakamura et al^21^ demonstrated higher needs for transfusion and endoscopic hemostasis. However, age can again be a confounder in these studies as patients on anti-thrombotics were older. Moreover, patients on anti-thrombotics presented with lower hemoglobin levels, which may have influenced the adverse outcome rates. In our study, patients with or without anti-thrombotics had similar ages and basal hemoglobin levels, and we did not see a difference in requirements for transfusion or endoscopic hemostasis. Similar basal hemoglobin levels in both groups may explain the similar need for transfusion. Importantly, increasing age may have more impact on the severity of UGIB than the use of anti-thrombotics.

In patients with UGIB under NSAIDS, earlier studies^22,23^ reported an increased risk of adverse events including re-bleeding and 30-day mortality; however, these findings were not reproduced in later studies.^24,25^ We also did not see a difference between NSAIDs and control groups for the transfusion, endoscopic treatment, re-bleeding, and 30-day mortality rates. An explanation for the improvement of the outcomes in newer studies may be the more widespread availability of endoscopic treatments which can decrease the rate and severity of complications of NSAIDs by allowing earlier diagnosis and management. We performed early endoscopy (within 24 hours) in all patients, which is associated with lower in-hospital mortality and less frequent re-bleeding in patients with NVUGIB.^8,26,27^

A peptic ulcer is known to be the most common cause of UGIB. Also, in our study, it was the most common cause of bleeding in all 3 groups. The frequency of peptic ulcer was higher in the NSAID group than in controls, which was consistent with the literature.^10^ In our study, the NSAID group also had a lower frequency of high-risk stigmata when compared to controls. This may be explained by the potential presence of an indolent ulcer in NSAIDs using patients until the condition becomes overt with UGIB. Opposite to our findings, Kim and colleagues^28^ reported increased high-risk stigmata with NSAIDs.

Stopping anti-thrombotic agents in NVUGIB reduces the risk of bleeding, but it increases the risk of thromboembolic events. European Society of Gastrointestinal Endoscopy^8^ and Asia-Pacific Working Group Consensus^29^ recommends that anti-thrombotics should not be discontinued in patients receiving anti-platelet therapy for secondary prophylaxis unless there is a high-risk stigmata finding (Forrest II c and III ulcers). When anti-thrombotics need to be stopped, they should be restarted as soon as the bleeding is controlled. If the patient is on anti-coagulants for atrial fibrillation or valvular heart disease, it is recommended to restart anti-thrombotics as soon as the bleeding is controlled, preferably within 7 days. In line with these guidelines, we held anti-thrombotics until the bleeding was controlled. All patients were also consulted to Cardiology or Pulmonology to individualize risk management. With this strategy, we have seen similar re-bleeding rates in patients with or without anti-thrombotics. However, in our study, thromboembolic events developed in 2 patients.

The strong side of our study is first its prospective design. Second, we performed an endoscopy on all patients which allowed us to better evaluate the etiology and characteristics of UGIB in study groups. There are several weaknesses in our study. We focused on determining the severity of bleeding in patients using anti-thrombotics, NSAIDs, or non-users. Since the study design was not epidemiological, we did not evaluate the risk of bleeding in using anti-thrombotics, NSAIDs, or non-users. We assessed LDA, anti-platelet, or anticoagulants as anti-thrombotics. Yamaguchi et al^16^ published a study with a similar design. Although studies have shown that the use of antiaggregant or anticoagulants comparably increases the risk of bleeding, this may have affected the study results. We excluded patients using both NSAIDs and anti-thrombotic drugs which is a common scenario, particularly in the older population. We also did not test the patients for *Helicobacter pylori*, which may have introduced a bias in the results.

In conclusion, NVUGIB increasingly occurs in older populations with several comorbidities; adverse outcomes are not associated with the use of NSAIDs or anti-thrombotics.

It is well known that those medication groups increase the bleeding risk; however, this is the first prospective study to evaluate their impacts on the clinical course. We have shown that NSAID, anti-platelet, or anti-coagulant use does not alter the prognosis, in older patients who presented with UGIB.

## Figures and Tables

**Table 1.** Patients’ Characteristics, Clinical Details, and Outcomes

	All Patients n = 257	No Drug n = 76 (30%)	NSAID n = 26 (10%)	Anti-Thrombotic n = 155 (60%)
Age, years	77.7 ± 8.2	77.5 ± 8.3	78.4 ± 8.0	77.7 ± 8.2
Male, n (%)	152 (59)	48 (63)	11 (42)	93 (60)
Comorbidities				
Heart diseases (AF, coronary heart disease, chronic heart failure), n (%)	160 (62)	27 (36)	7 (27)	126 (81)^a^
Hypertension, n (%)	158 (61)	36 (47)	13 (50)	109 (70)
Cerebrovascular diseases, n (%)	28 (11)	6 (8)	0 (0)	22 (14)
Diabetes mellitus, n (%)	69 (27)	14 (18)	7 (27)	48 (31)
Chronic renal disease, n (%)	41 (16)	14 (18)	4 (15)	23 (15)
Malignant diseases, n (%)	38 (15)	18 (24)	5 (19)	15 (10)
PPI use	83 (32)	33 (43)	8 (28)	42 (27)
Previous episode of UGIB, n (%)	56 (22)	20 (26)	5 (19)	31 (20)
Hemoglobin level on admission (g/dL), n (%)	9.3 ± 2.7	9.4 ± 2.6	9.5 ± 2.8	9.2 ± 2.7
Urea level on admission (mg/dL), n (%)	92.0 (55.5-141.0)	89.0 (55.0-151.0)	95.0 (54.0-131.0)	92.0 (56.0-141.0)
Serum albumin level on admission (g/L), n (%)	34.5 ± 6.7	33.2 ± 5.5	34.4 ± 5.5	33.4 ± 7.3
Glasgow Blatchford score ≤1	5 (2)	1 (1)	0 (0)	4 (3)
AIMS65 score “0”	6 (2)	3 (4)	0 (0)	3 (2)
Glasgow Blatchford score >1	255 (98)	75 (98)	26 (100)	152 (98)
AIMS65 score ≥2	124 (48)	36 (47)	8 (30)	80 (52)
Clinical outcomes				
Need for blood transfusion, n (%)	167 (65)	44 (58)	17 (65)	106 (68)
Need for endoscopic hemostasis, n (%)	78 (30)	24 (32)	5 (19)	49 (32)
Need for surgery/interventional radiology, n (%)	7 (3)	4 (5)	1 (4)	2 (1)
Hospitalization, n (%)	193 (75)	54 (71)	21 (81)	118 (76)
Re-bleeding (during hospitalization), n (%)	25 (10)	8 (11)	0 (0)	17 (11)
30-day mortality, n (%)	40 (16)	17 (22)	4 (15)	19 (12)

. Results are expressed as mean ± SD or median (IQR) or frequency (%). Incidence of heart disease was significantly higher in patients using anti-thrombotics than in patients who did not use drugs (^a^ *P* < .001).

AF, atrial fibrillation; NSAIDs, non-steroidal anti-inflammatory drugs; UGIB, upper gastrointestinal bleeding.

**Table 2.** Endoscopic Findings and Details of Those Using Anti-Thrombotic Drugs or NSAIDs or Neither

	No Drug n = 76 (30%)	NSAID n = 26 (10%)	Anti-Thrombotics n = 155 (60%)
Peptic ulcer, n (%)	36 (47)	18 (69)^a^	70 (45)
Gastric, n (%)	12 (16)	8 (31)	30 (19)
Upper	3 (4)	3 (12)	3 (2)
Middle	8 (11)	3 (12)	17 (11)
Lower	13 (17)	4 (15)	21 (14)
Duodenal, n (%)	24 (32)	10 (39)	40 (26)
Number of ulcer, n (%)			
Single	36 (47)	16 (62)	72 (47)
Multiple	9 (12)	6 (4)	10 (7)
Size of ulcer (mm), n (%)			
<10	22 (29)	9 (35)	46 (30)
10-20	15 (20)	7 (27)	26 (17)
>20	9 (12)	6 (23)	10 (7)
Forrest, n (%)			
Ia, Ib, IIa, IIb	16 (21)	5 (19)	37 (24)
IIc, III	28 (37)	17 (65)^b^	45 (29)
Erosive esophagitis, n (%)	3 (4)	1 (4)	5 (3)
Esophageal ulcer, n (%)	7 (9)	0 (0)	7 (5)
Upper gastrointestinal malignancy, n (%)	8 (11)	2 (8)	11 (7)
Erosive/hemorrhagic gastropathy- duodenopathy, n (%)	9 (12)	2 (8)	34 (22)
Angioectasia, n (%)	7 (9)	1 (4)	7 (5)
Dieulafoy lesion, n (%)	1 (1)	0 (0)	0 (0)
Lesion not visualized, n (%)	2 (3)	0 (0)	11 (7)

The incidence of peptic ulcer was significantly higher in patients using NSAID than in patients who did not use drugs (^a^ *P* = .05). According to the Forrest classification, patients with IIc and III ulcers were statistically significantly higher in patients using NSAID than no used drugs (^b^ *P* = .01).

NSAID, non-steroidal anti-inflammatory drugs.
